# Is neuroendocrine differentiation a prognostic factor in poorly differentiated colorectal cancer?

**DOI:** 10.1186/s12957-017-1139-y

**Published:** 2017-03-28

**Authors:** Yue Chen, Fang Liu, Qingkai Meng, Siping Ma

**Affiliations:** Department of colorectal surgery, Cancer Hospital of China Medical University, Liaoning Cancer Hospital & Institute, No. 44 Xiaoheyan Road, Dadong District, Shenyang, 110042 Liaoning Province People’s Republic of China

**Keywords:** Poorly differentiated colorectal cancer, Neuroendocrine differentiation, Lymph node metastasis, Prognosis

## Abstract

**Background:**

To determine the prognostic relevance of neuroendocrine differentiation in poorly differentiated colorectal cancer.

**Methods:**

The clinicopathological features and survival of 70 patients with poorly differentiated colorectal cancer were analyzed retrospectively. Chromogranin A and synaptophysin were used as neuroendocrine markers. Patients were followed-up for more than 3 years or until death.

**Results:**

Of these 70 patients, 36 showed neuroendocrine differentiation. In univariate prognostic analysis, the patients with lymph node metastasis (*P* < 0.001), advanced TNM stage (*P* < 0.001), and neuroendocrine differentiation (*P* = 0.003) tended to have a poor prognosis. However, only lymph node metastasis was associated with a poor prognosis in multivariate analysis (*P* < 0.001). Patients with neuroendocrine differentiation were associated with lymph node metastasis (*P* = 0.006).

**Conclusions:**

Neuroendocrine differentiation in poorly differentiated colorectal cancer was not a direct prognostic factor in these patients. Lymph node metastasis was a direct prognostic factor in these patients. Patients with neuroendocrine differentiation were associated with lymph node metastasis.

## Background

Neuroendocrine differentiation (NED) has been observed in cancers of several non-neuroendocrine organs, including the gastrointestinal tract. Many studies have evaluated the clinical prognostic value of NED in colorectal cancer (CRC). However, conflicting data exist in these studies. In the study by Mori et al. [1], NED did not influence patient prognosis. Similarly, Lloyd et al. [2] showed that, in 289 patients, NED did not influence prognosis in moderately differentiated colorectal carcinomas. In contrast, in recent years, studies have revealed that NED does influence patient prognosis. Bernick et al. [3] studied 38 CRC patients with NED and found that the prognosis of these patients was poor. Similar findings were reported in a meta-analysis by Zeng et al. [4]. Intriguingly, some studies have indicated that CRC with NED was correlated with liver metastasis and advanced tumor stages [3, 5].

Poorly differentiated colorectal cancer (PDCRC) comprises 5 to 25% of all CRCs [6–9]. It is known that PDCRC is usually associated with a poor prognosis. NED is often found in PDCRC [5]. The prognosis of PDCRC with NED is currently still unclear. Here, we preliminarily studied whether NED was a prognostic factor in patients with PDCRC.

## Methods

### Patients

Between 2008 and 2012, 70 patients with primary PDCRC who underwent radical resection were analyzed retrospectively. All patients with TNM II and III tumors, according to the 7th edition of the American Joint Committee on Cancer/Union International control Cancer (AJCC/UICC) tumor-node-metastasis (TNM) staging system, received adjuvant therapy after surgery. Histology specimens were evaluated by two senior pathologists, and the diagnosis of PDCRC was confirmed in all patients. Patients who died perioperatively and those with distant organ metastasis or secondary malignancy were excluded.

### Immunohistochemistry

Hematoxylin and eosin-stained specimens from each patient were available for review. In addition, each specimen was analyzed for the biological markers of NED, synaptophysin (Syn), and chromogranin A (CgA) by immunohistochemistry. The number of Syn or CgA immunoreactive cells was determined using an eyepiece at high-power field (HPF). Immunoreactive cells were counted in at least ten most concentrated areas of tumor cells, and the results were presented as the mean number of immunoreactive cells per HPF. When no immunoreactive tumor cells for CgA and Syn were noted in all tumor fields, the tumor was classified as NED(−). Moreover, when ≥1 tumor cells/HPF were positive for CgA and/or Syn, the tumor was classified as NED(+) [10–12]. Furthermore, NED(+) tumors were assigned to three subgroups based on the presence of immunoreactive cells per HPF: subgroup 1(SG1) had less than 10% immunoreactive cells of the total number of cells per HPF; subgroup 2(SG2) had 10–20% immunoreactive cells; subgroup 3(SG3) had more than 20% immunoreactive cells. According to the 2010 World Health Organization (WHO) classification, specimens with more than 30% immunoreactive cells were classified as mixed adenoneuroendocrine carcinoma (MANEC), and these patients were excluded from the study.

### Statistics

All statistical analyses were performed using SPSS (version 16.0). Pearson’s chi-squared test was used to investigate correlations of clinicopathological features between the NED(+) and NED(−) groups. Multivariate analysis was performed using logistic regression. Overall survival was assessed by the Kaplan-Meier method. Statistical significance between the survival curves of clinicopathological features was calculated by the log rank test. Significant variables were then examined by multivariate analysis using the Cox model. *P* values < 0.05 were considered statistically significant.

## Results

### General information

Seventy patients (36 males and 34 females) were diagnosed with PDCRC, including 36 (51.4%) NED(+) patients and 34 (48.6%) NED(−) patients. The mean age was 60.2 years (range: 36–86 years). Of these patients, 2 had stage I cancer, 17 had stage II, and 51 had stage III, which included 45 cases of colon cancer and 25 cases of rectal cancer. The median survival time of these patients was 47.656 months. Thirty-nine of 70 patients died during the follow-up period.

### Prognostic factors of PDCRC

Survival analyses were based on 70 patients with complete follow-up data. Overall mean survival time was 47.656 months (95% confidence interval: 38.557–56.755) and 3-year overall survival (3-year OS) was 0.469 ± 0.060.

**Fig. 1:**
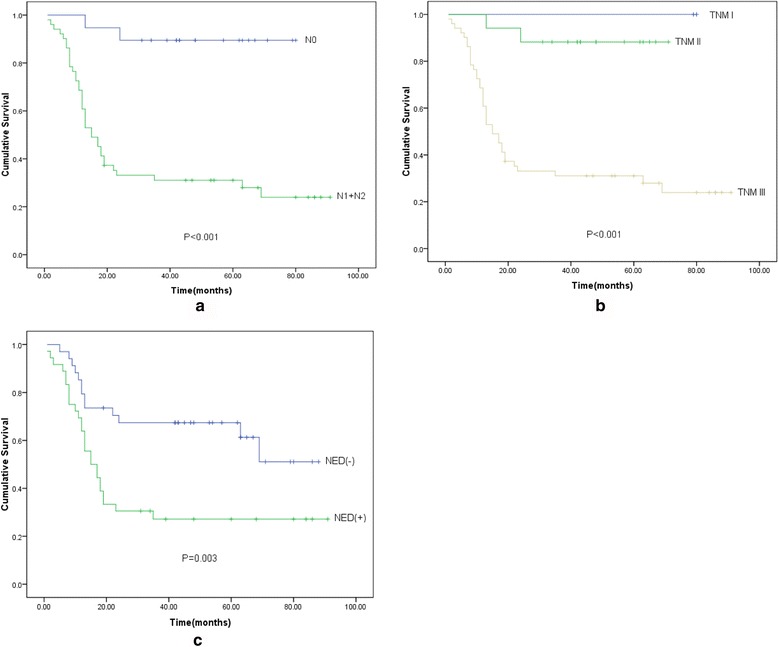
Prognostic analysis of clinicopathological factors for overall survival in PDCRC. **a** Kaplan-Meier survival analysis of N0 and N1 + N2 patients. The patients with N stage(+) tended to have a poor prognosis (*P* < 0.001). **b** Kaplan-Meier survival analysis of TNM stage I, II, and III patients. The patients with advanced TNM stage tended to have a poor prognosis (*P* < 0.001). **c** Kaplan-Meier survival analysis of NED(−) and NED(+) patients. The patients with NED(+) tended to have a poor prognosis (*P* = 0.003)

In these PDCRC cases, the patients with N stage(+) (*P* < 0.001), advanced TNM stage (*P* < 0.001), and NED(+) (*P* = 0.003) tended to have a poor prognosis(Fig. 1). However, there was no significant difference in 3-year OS in terms of age, gender, tumor location, lymph nodes retrieved ,and T stage (Table 1).

**Table 1:** Prognostic analysis of clinicopathological factors for overall survival in poor differentiated colorectal cancer patients

Clinicopathological factors	Univariate analysis			Multivariate analysis	
	Number(%)	3-year OS	*P* value	*P* value	RR
Age			0.330		
<65 years	43(61.4%)	0.510 ± 0.077			
≥65 years	27(38.6%)	0.407 ± 0.095			
Gender			0.582		
Male	36(51.4%)	0.408 ± 0.083			
Female	34(48.6%)	0.529 ± 0.086			
Tumor location			0.092		
Colon	45(64.3%)	0.503 ± 0.083			
Rectum	25(35.7%)	0.315 ± 0.094			
T stage			0.373		
T1 + T2	5(7.1%)	0.600 ± 0.219			
T3 + T4	65(92.9%)	0.459 ± 0.062			
N stage			<0.001*	<0.001*	3.028
N0	19(27.1%)	0.895 ± 0.070			
N1 + N2	51(72.9%)	0.310 ± 0.065			
TNM stage			<0.001*	0.384	
I	2(2.9%)	NA^a^			
II	17(24.3%)	0.882 ± 0.078			
III	51(72.8%)	0.310 ± 0.065			
NED			0.003*	0.111	
+	36(51.4%)	0.272 ± 0.075			
-	34(48.6%)	0.674 ± 0.081			

*Indicated statistical significance (*P* < 0.05)

^a^Not applicable

In multivariate analysis using the Cox model, only lymph node metastasis (LNM) was associated with a poor prognosis (*P* < 0.001) (Table 1).

**Fig. 2:**
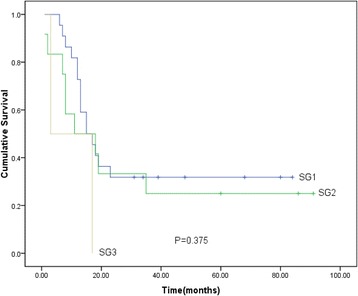
Prognostic analysis of SGs 1, 2, and 3 for overall survival in NED(+) patients. There were no significant differences among three subgroups (*P* = 0.375)

Moreover, by stratification analysis based on the degree of NED, we analyzed the prognosis of SGs1, 2, and 3. Interestingly, with the increasing presence of immunoreactive cells, the subgroup with higher expression tended to have a poor prognosis. However, this trend did not reach statistical significance (Fig. 2).

### Clinicopathological features and neuroendocrine differentiation

Univariate analysis revealed that N stage (*P* = 0.010) and TNM stage (*P* = 0.018) had correlation with NED. Furthermore, in multivariate analysis, only N stage was associated with NED (*P* = 0.006). There was no significant association between NED and age, gender, tumor location, lymph nodes retrieved, and T stage (Table 2).

**Table 2:** Correlation between clinicopathological factors and neuroendocrine differentiation

Clinicopathological factors	Number(%)	NED		Univariate analysis	Multivariate analysis	
		+(SG1, SG2, SG3)	–		*P* value	OR
Age				0.955		
<65 years	43(61.4%)	22(15, 6, 1)	21			
≥65 years	27(38.6%)	14(7, 6, 1)	13			
Gender				0.816		
Male	36(51.4%)	19(14, 4, 1)	17			
Female	34(48.6%)	17(8, 8, 1)	17			
Tumor location				0.943		
Colon	45(64.3%)	23(13, 9, 1)	22			
Rectum	25(35.7%)	13(9, 3, 1)	12			
Lymph nodes retrieved				0.492		
<12	18(25.7%)	8(6, 1, 1)	10			
≥12	52(74.3%)	28(16, 11, 1)	24			
T stage				0.669^a^		
T1 + T2	5(7.1%)	2(2, 0, 0)	3			
T3 + T4	65(92.9%)	34(20, 12, 2)	31			
N stage				0.010*	0.006*	2.391
N0	19(27.1%)	5(5, 0, 0)	14			
N1 + N2	51(72.9%)	31(17, 12, 2)	20			
TNM stage				0.018^b,^*	0.755	
I	2(2.9%)	0(0, 0, 0)	2			
II	17(24.3%)	5(5, 0, 0)	12			
III	51(72.8%)	31(17, 12, 2)	20			

^a^Fisher’s exact test

^b^Likelihood radio

*Indicated statistical significance (*P* < 0.05)

## Discussion

In a previous study, the incidence of NED in PDCRC was 41.5% [12]. In our study, the incidence was 51.4%, which was close to that in Liu’s study. Since NED is quite common in PDCRC, it is important to study the influence of NED in PDCRC patients.

There is controversy regarding the prognostic significance of NED in CRC [1–4, 13–15]. In our study, there were statistically significant differences in 3-year OS in terms of N stage, TNM stage, and NED in patients with PDCRC. Multivariate analysis indicated that N stage(+) was a significant negative prognostic factor for 3-year OS, which was not surprising as this parameter is a well-known marker with prognostic relevance [16–19]. In a previous study, de Bruine et al. [14] suggested that adenocarcinomas with NED tended to exhibit early LNM, which was similar to the results in our correlation analysis. We found that patients with NED were associated with LNM. Therefore, it may be deduced that NED, which might influent LNM, affected the prognosis of patients with PDCRC, rather than being a direct prognostic factor in these patients. In many studies, there were differences in the proportion of patients with LNM due to small sample sizes [1–4, 13–15]. Therefore, this may be the reason why there is controversy regarding the prognostic significance of NED in CRC. Moreover, the biological mechanisms underlying PDCRC with NED and metastasis remain unclear. It is known that neuroendocrine differentiated cells secrete neurohormonal substances by the autocrine or paracrine loop. In a previous study, biogenic amines and polypeptide hormones played a role in the growth regulation of normal and neoplastic intestinal epithelia [20]. Hypothetically, NED could stimulate growth and metastatic capacity through the secretion of neurohormonal substances.

Moreover, by further stratified analysis, we found that prognoses of three subgroups did not reach statistical significance. But, the subgroup with higher expression tended to have a poor prognosis. This might be due to small sample size in our study. Therefore, further investigations are required to confirm this hypothesis.

TNM staging is a classic staging method used to predict survival. However, with the development of molecular medicine, TNM staging may be less reliable in predicting survival, as shown in the research by Eschrich et al. They argued that molecular staging might provide an accurate prognosis for cancer patients [21]. Our data indicated that patients with NED were associated with LNM. Therefore, NED may be an important factor in molecular staging. Several studies had indicated that the neuroendocrine phenotype was associated with increased chemosensitivity in lung cancer [22, 23] and in colorectal cell lines [24]. Therefore, NED might also be helpful in the therapy of CRC. Further investigations are required in this area.

## Conclusions

NED is a common event in PDCRC. NED in PDCRC is not a direct prognostic factor in these patients. LNM is a direct prognostic factor in these patients. Patients with NED were associated with LNM. Moreover, NED, which might influent LNM, affected the prognosis of patients with PDCRC. Further research on this important issue is required.

## Abbreviations

3-year OS: 3-year overall survival
AJCC/UICC: American Joint Committee on Cancer/ Union for International Cancer Control
CgA: Chromogranin A
CRC: Colorectal cancer
HPF: High-power field
LNM: Lymph node metastasis
MANEC: Mixed adenoneuroendocrine carcinoma
NED: Neuroendocrine differentiation
PDCRC: Poorly differentiated colorectal cancer
SG1: Subgroup 1
SG2: Subgroup 2
SG3: Subgroup 3
Syn: Synaptophysin
TNM: Tumor-node-metastasis
WHO: World Health Organization
